# Comparison of the Non‐intubated Behavioral Pain Scale Score During Replacement of Ideal Button ZERO and Non‐ZERO Gastrostomy Tubes

**DOI:** 10.1002/deo2.70254

**Published:** 2025-11-27

**Authors:** Kazumi Shimamoto, Masanori Hongo, Naoki Dan, Naoko Mori, Yu Kobayashi, Hiromitsu Ban

**Affiliations:** ^1^ Department of Gastroenterology Omi Medical Center Kusatsu City Japan

**Keywords:** complication, endoscopy, gastrostomy, pain, percutaneous

## Abstract

**Objective:**

Many patients report pain during bumper‐type gastrostomy tube replacement. The Ideal Button ZERO (ZERO) is a 24‐Fr bumper‐type gastrostomy tube that was developed to eliminate such pain, but its efficacy has not yet been evaluated. We compared the pain during replacement of the ZERO and of other non‐ZERO bumper‐type gastrostomy tubes.

**Methods:**

This retrospective cohort study included 180 patients who underwent gastrostomy tube replacement at our institution during 2023–2024. The primary outcome was the Non‐intubated Behavioral Pain Scale (BPS‐NI) score when the gastrostomy tubes were replaced. Scores were compared among ZERO, non‐ZERO 20‐Fr, and non‐ZERO 24‐Fr groups.

**Results:**

Pain during gastrostomy tube removal was assessed in 80 (44.4%), 66 (36.7%), and 34 (18.9%) patients in the ZERO, non‐ZERO 20‐Fr, and non‐ZERO 24‐Fr groups, respectively. The median BPS‐NI was significantly lower in the ZERO (3.0 [interquartile range [IQR]: 3.0–3.0]) than non‐ZERO 20‐Fr (6.0 [IQR: 4.0–8.0]) and non‐ZERO 24‐Fr (5.0 [IQR: 4.0–7.0]) groups. Pain during gastrostomy tube insertion was assessed as follows: the ZERO was replaced from a 24‐Fr tube and a 20‐Fr tube in 80 (44.4%) and 41 (22.8%) cases, respectively; a non‐ZERO 20‐Fr tube and a non‐ZERO 24‐Fr tube were inserted in 25 (13.9%) and 34 (18.9%) cases, respectively. The median BPS‐NI score was significantly lower in the ZERO (replaced 24‐Fr) group (3.0 [IQR: 3.0–3.3]) than in both the non‐ZERO 20‐Fr (5.0 [IQR: 3.0–6.0]) and non‐ZERO 24‐Fr (5.0 [IQR: 4.0–6.0]) groups.

**Conclusions:**

Use of the ZERO reduces pain during gastrostomy tube replacement.

## 1 Introduction

Percutaneous endoscopic gastrostomy (PEG), developed by Gauderer et al. [1] in 1980, is a widely used method of gaining gastrointestinal access for providing artificial nutrition. Gastrostomy tubes have two types of internal bolsters: the balloon‐type and the bumper‐type. Compared with balloon‐type and bumper‐type tubes have the advantage of reducing the frequency of gastrostomy tube replacement; however, bumper‐type tubes may cause severe pain during their insertion and removal, as well as bleeding from the stoma.

**FIGURE 1:**
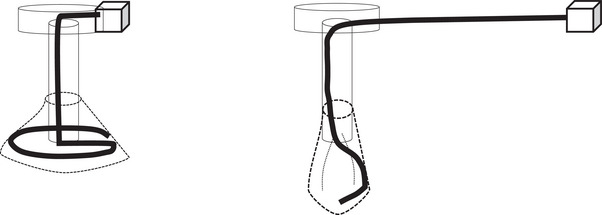
Procedure for removing the ZERO. Pulling the shape‐memory wire out of the external bolster section causes the bumper to become a silicone bag.

**FIGURE 2:**
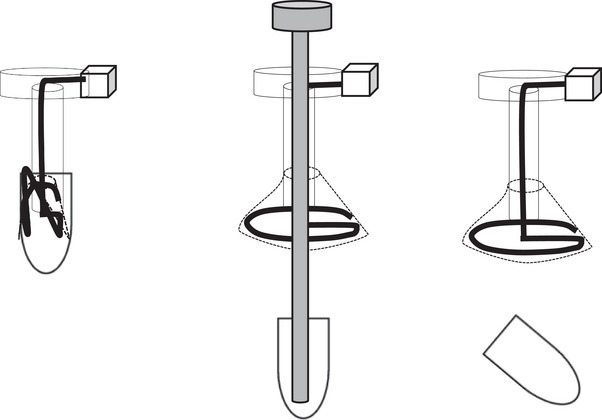
Procedure for inserting the ZERO. Pushing the capsule surrounding the bumper into the stomach causes the shape‐memory wire to expand into the silicone bag, forming the bumper.

The Ideal Button ZERO (hereafter, ZERO), a bumper‐type gastrostomy tube, was developed to reduce snagging of the bumper during tube insertion and removal, thereby decreasing patient discomfort; however, no reports to date have investigated its efficacy. The ZERO bumper consists of a silicone bag and shape‐memory wire (nickel–titanium alloy). When the gastrostomy tube is removed, pulling the shape‐memory wire out from the external bolster section causes the bumper to become a shapeless silicone bag (Figure 1), allowing for smooth removal. During its insertion, pushing the capsule surrounding the bumper into the stomach causes the shape‐memory wire to expand the silicone bag, forming the bumper (Figure 2). The capsule has a diameter of 9.3 mm, only 1.3 mm larger than the stoma (shaft) diameter (8 mm; 24 Fr), and is composed of gelatin that dissolves in the stomach. To the best of our knowledge, no reports to date have evaluated whether the use of the ZERO reduces patient discomfort. Knowing whether the ZERO reduces discomfort during gastrostomy tube replacement would be highly beneficial for physicians and for patients requiring PEG procedures.

In this study, we conducted an objective evaluation of the pain experienced during gastrostomy tube replacement in patients who underwent PEG, and set out to determine whether the ZERO caused less pain than did other gastrostomy tubes.

## 2 Methods

### 2.1 Study Participants

This study included all patients who underwent bumper‐type gastrostomy tube replacement after PEG at the Omi Medical Center between February 2023 and June 2024. Patients who underwent balloon‐type gastrostomy tube replacement, or gastrostomy using methods other than PEG, were excluded. No patients were sedated during tube replacement.

All experimental protocols described in this study were approved by the Institutional Ethical Review Committee of the Omi Medical Center (authorization number: 2025‐0055). The study adhered to the ethical guidelines of the Japan Ministry of Health, Labor, and Welfare for Medical and Health Research Involving Human Subjects, and conformed to the principles outlined in the Declaration of Helsinki. The opt‐out recruitment method was used, allowing patients to decline participation; informed consent was thus obtained from all participants.

### 2.2 Bumper‐type Gastrostomy Tubes Used in this Study

The non‐ZERO bumper‐type gastrostomy tubes used as comparators in this study were the Endovibe Button II (20‐Fr, 24‐Fr; Boston Scientific, Marlborough, MA, USA) and Ideal Button (20‐Fr, 24‐Fr; Olympus, Tokyo, Japan).

### 2.3 Confirmatory Imaging During Replacement

We use either an X‐ray or an endoscope to ensure that the gastrostomy tube is securely placed in the stomach. Basically, we use X‐ray and endoscope alternately at each 6‐month replacement.

### 2.4 Pain Score

The Non‐intubated Behavioral Pain Scale (BPS‐NI) was used to score pain on a scale of 3 (no pain) to 12 (maximal pain) [2, 3]. The pain assessments were conducted by a single nurse.

### 2.5 Outcome

The primary outcomes were the BPS‐NI score at gastrostomy tube insertion and removal in the ZERO and non‐ZERO groups.

### 2.6 Statistical Analyses

Fisher's exact test was used to compare patient demographics between the two groups, logistic regression analysis was used to identify factors associated with the BPS‐NI, and the Kruskal–Wallis test was used to analyze overall group comparisons, and the Steel–Dwass test was used to assess between‐group comparisons in pain scores. All statistical analyses were performed using EZR software (Saitama Medical Center, Jichi Medical University, Japan). Statistical significance was set at *p* < 0.05.

## 3 Results

### 3.1 Patient Demographics

The patient demographics are summarized in Table 1. Of the 180 patients who underwent gastrostomy tube removal in this study, 80 (44.4%) and 100 (55.6%) were categorized into the ZERO and non‐ZERO groups, respectively. Age‐related changes were significantly more common in the ZERO group during gastrostomy tube removal as a cause of dysphagia. In the patient group undergoing endoscopy, the endoscope remained inserted throughout the procedure.

**TABLE 1:** Characteristics of study patients.

	BPS‐NI at gastrostomy tube removal			BPS‐NI at gastrostomy tube insertion		
	**ZERO** **(*n* = 80)**	**Non‐ZERO** **(*n* = 100)**	***p*‐value**	**ZERO** **(*n* = 121)**	**Non‐ZERO** **(*n* = 59)**	***p*‐value**
Age (years), mean ± SD	80.39 ± 10.51	79.51 ± 10.05	0.569	80.40 ± 10.1	78.88 ± 10.54	0.353
Sex Male	31 (38.8%)	37 (37.0%)	0.877	49 (40.5%)	19 (32.2%)	0.327
Female	49 (61.3%)	63 (63.0%)		72 (59.5%)	40 (67.8%)	
BMI (kg^/^m^2^), mean ± SD	17.59 ± 2.71	17.98 ± 2.93	0.355	17.77 ± 2.79	17.88 ± 2.94	0.814
PS, median [IQR]	3.00 [3.00, 4.00]	3.00 [3.00, 4.00]	0.717	3.00 [3.00, 4.00]	3.00 [3.00, 4.00]	
MNA‐SF, median [IQR]	8.00 [6.00, 9.00]	7.00 [5.00, 9.00]	0.948	8.00 [6.00, 9.00]	7.00 [5.00, 9.00]	
**Background (cause of dysphagia)**						
CVD	37 (46.2%)	59 (59.0%)	0.100	60 (49.6%)	37 (62.7%)	0.112
ND	13 (16.2%)	19 (19.0%)	0.697	21 (17.4%)	11 (18.6%)	0.838
TBI	5 (6.2%)	3 (3.0%)	0.469	7 (5.8%)	1 (1.7%)	0.276
Tumor (Neck cancer, Neurogenic tumor, Lung cancer)	8 (10.0%)	8 (8.0%)	0.793	9 (7.4%)	6 (10.2%)	0.571
Age‐related change	15 (18.8%)	8 (8.0)	0.043	19 (15.7%)	4 (6.8%)	0.102
Dementia	2 (2.5%)	3 (3.0%)	1.000	5 (4.1%)	0 (0%)	0.174
**Confirmatory imaging during replacement**						
Endoscopy	45	57	1.000	66	36	1.000
X‐ray	35	43		55	23	

*SD* standard deviation, *BMI* body mass index, *IQR* interquartile range, *PS* performance status, *MNA‐SF* Mini Nutritional Assessment Short‐Form, *CVD* cerebrovascular disease, *ND* neurodegenerative disease, *TBI* traumatic brain injury, *PEG* percutaneous endoscopic gastrostomy, *BPS‐NI* Non‐intubated Behavioral Pain Scale.

### 3.2 Binary Logistic Regression Analysis to Identify Independent Factors Associated With BPS‐NI (Cut Off 5) During Gastric Tube Removal

We investigated independent factors affecting BPS‐NI. We divided patients into those with BPS‐NI≤4(moderate or less pain) and those with BPS‐NI ≥ 5 (severe pain) [2], and the effects of age, sex, BMI, endoscope use or not, and non‐ZERO use or ZERO use were analyzed using logistic regression analysis. ZERO or non‐ZERO was the only factor that affected BPS‐NI at tube removal (odds ratio, 20.90; 95% confidence interval [95%CI], 8.13–53.9; *p* < 0.001) (Table 2). Compared to the ZERO insertion replaced from 24‐Fr, the insertion of non‐ZERO 20‐Fr, non‐ZERO 24‐Fr, and ZERO replaced from 20‐Fr was a significantly influencing factor that affected BPS‐NI (odds ratio, 9.03; 95%CI, 2.65–30.8; *p* < 0.001, odds ratio, 8.70; 95%CI, 3.37–22.50; *p* < 0.001, odds ratio, 19.40; 95%CI, 7.31–51.50; *p* < 0.001) (Table 2).

**TABLE 2:** Binary logistic regression analysis to identify independent factors associated with Non‐intubated Behavioral Pain Scale (BPS‐NI) (cut off 5) during gastrostomy tube replacement.

	During gastrostomy tube removal			During gastrostomy tube insertion			
	OR	95%CI	*p‐*value		OR	95%CI	*p‐*value
Age (years)	0.99	0.95–1.03	0.59	Age (years)	1.01	0.97 to 1.05	0.68
Sex (Male)	0.57	0.24–1.32	0.19	Sex (Male)	0.79	0.35 to 1.78	0.56
BMI (kg/ m^2^)	1.03	0.90–1.18	0.68	BMI (kg/m^2^)	1.00	0.88 to 1.14	0.99
Use of endoscopy	1.12	0.51–2.46	0.77	Use of endoscopy	1.39	0.66 to 2.96	0.39
Use of non‐ZERO	20.90	8.13–53.9	<0.001	Zero replaced from 24Fr vs non‐ZERO20 Fr	9.03	2.65 to 30.8	<0.001
				Zero replaced from 24Fr vs non‐ZERO 24Fr	8.70	3.37 to 22.50	<0.001
				Zero replaced from 24Fr vs Zero replaced from 20Fr	19.40	7.31 to 51.50	<0.001

*OR* odds ratio, *CE* Coefficient estimate, *CI c*onfidence interval, *BMI* body mass index.

### 3.3 Comparison of BPS‐NI

**FIGURE 3:**
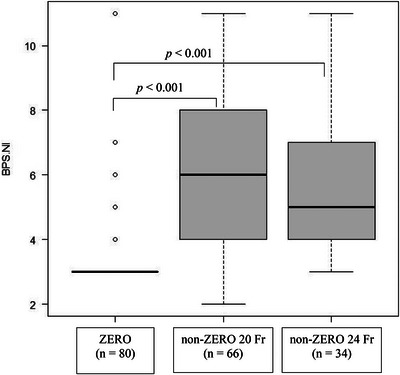
Comparison of BPS‐NI scores during gastrostomy tube removal. BPS‐NI, Non‐intubated Behavioral Pain Scale.

All patients underwent pain assessment during gastrostomy tube removal. Patients in the non‐ZERO group were classified based on the type of gastrostomy tube used: non‐ZERO 20‐Fr (66/180 patients, 36.7%), non‐ZERO 24‐Fr (34/180 patients, 18.9%). At tube removal, BPS‐NI scores differed across the three groups (Kruskal‐Wallis test, *p* < 0.001). The median BPS‐NI score at tube removal was significantly lower in the ZERO group (3.0 interquartile range [IQR]: 3.0–3.0) than in the non‐ZERO 20‐Fr (6.0 [IQR: 4.0–8.0]) and non‐ZERO 24‐Fr (5.0 [IQR: 4.0–7.0]) groups (Steel‐Dwass test, *p* < 0.001 for both comparisons) (Table 3 and Figure 3).

**TABLE 3:** Non‐intubated Behavioral Pain Scale (BPS‐NI) during gastrostomy tube removal and insertion.

	Gastrostomy tube removal		
During removal	ZERO *n* = 80	Non‐ZERO 20‐Fr *n* = 66	Non‐ZERO 24‐Fr *n* = 34
BPS‐NI Median (IQR)	3 (3.0–3.0)	6 (4.0–8.0)	5 (4.0–7.0)
Average (SD)	3.43 (1.12)	6.12 (2.41)	5.45 (2.37)
95% CI	3.175–3.675	5.484–6.761	4.613–6.296

	Gastrostomy tube insertion			
During insertion	ZERO Replaced 24‐Fr *n* = 80	Non‐ZERO 20‐Fr *n* = 25	Non‐ZERO 24‐Fr *n* = 34	ZERO Replaced 20‐Fr *n* = 41
BPS‐NI Median (IQR)	3 (3.0–3.3)	5 (3.0–6.0)	5 (4.0–6.0)	6.5 (4.75–8.25)
Average (SD)	3.55 (1.23)	5.47 (2.58)	5.39 (2.17)	6.60 (2.27)
95% CI	3.276–3.824	4.146–6.795	4.621–6.166	5.873–7.327

The Zero's shaft diameter is 24 Fr. BPS‐NI, Non‐intubated Behavioral Pain Scale; IQR, interquartile range; SD, Standard Deviation; CI, Confidence Interval.

**FIGURE 4:**
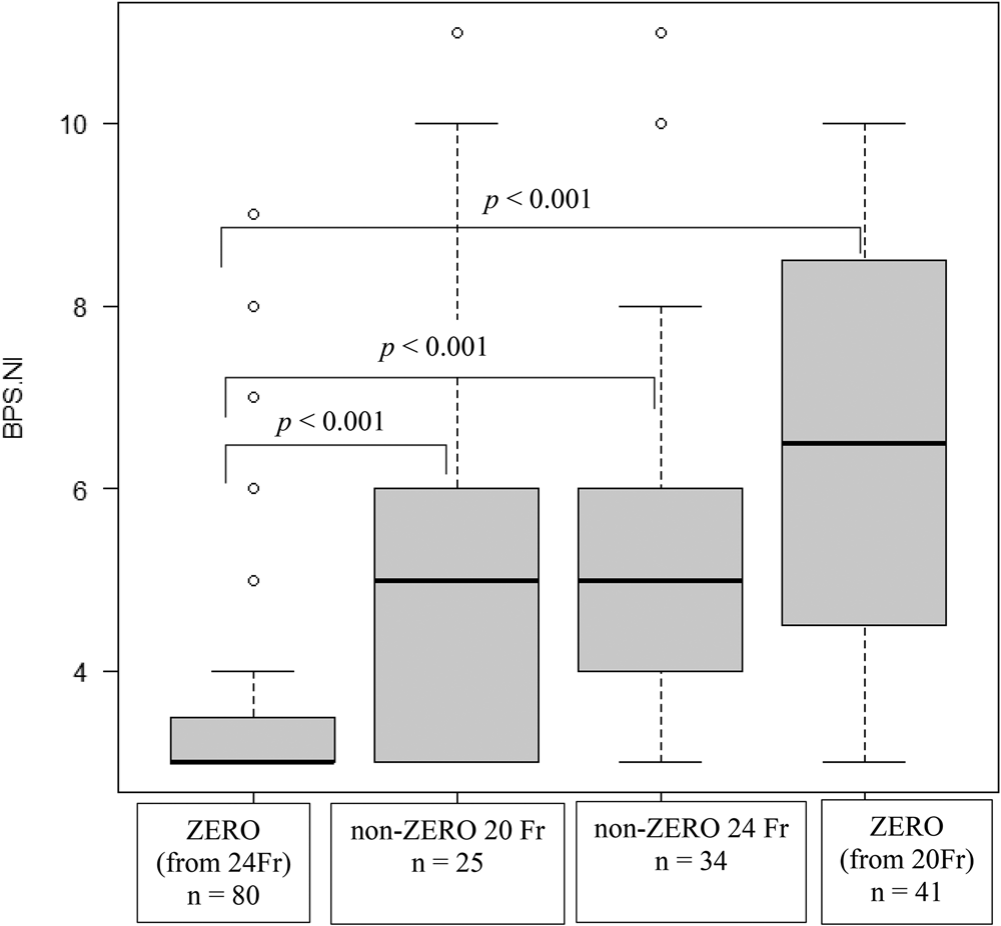
Comparison of BPS‐NI scores during gastrostomy tube insertion. BPS‐NI, Non‐intubated Behavioral Pain Scale.

Pain during gastrostomy tube insertion was assessed in all patients in the following contexts: replaced a 24‐Fr tube with ZERO in 80 (44.4%) cases, replaced a 20‐Fr tube with ZERO in 41 (22.8%) cases; a non‐ZERO 20‐Fr tube was inserted in 25 (13.9%) cases, and a non‐ZERO 24‐Fr tube was inserted in 34 (18.9%) cases. At tube insertion, BPS‐NI scores differed across the four groups (Kruskal‐Wallis test, *p* < 0.001). The median BPS‐NI score during tube insertion was significantly lower in the ZERO (replaced from 24‐Fr; 3.0 [IQR: 3.0–3.3]) group than in the non‐ZERO 24‐Fr (5.0 [IQR: 4.0–6.0]) and non‐ZERO 20‐Fr (5.0 [IQR: 3.0–6.0]) groups (Steel‐Dwass test, *p* < 0.001 for both comparisons) (Table 3 and Figure 4). However, the median BPS‐NI score of the ZERO (replaced from 20‐Fr) group (6.5 [IQR: 4.75–8.25]) was not significantly different from that in the non‐ZERO 24‐Fr (5.0 [IQR: 4.0–6.0]) and non‐ZERO 20‐Fr (5.0 [IQR: 3.0–6.0]) groups (Steel‐Dwass test, *p* = 0.098; *p* = 0.28, for both comparisons) (Table 3 and Figure 4).

### 3.4 Complications

There were no serious complications associated with gastrostomy tube replacement in all patients. Fistula bleeding occurring during replacement ZERO to ZERO was 0/69 (0%) cases, while fistula bleeding was observed in 17/111 (15.3%) cases using non‐ZERO. All hemorrhages were stopped by pressure application.

## 4 Discussion

In this study, we compared the BPS‐NI scores to assess pain during replacement of the ZERO and non‐ZERO gastrostomy tubes. No previous study has revealed the effect of using the ZERO on pain during gastronomy, the insertion or removal. We demonstrated that using the ZERO reduced patient discomfort. Several new findings emerged from this study; first, the BPS‐NI score during removal or insertion of the ZERO (replaced 24‐Fr) was significantly lower than that of the other bumper‐type gastrostomy tubes. Second, pain was not alleviated when the ZERO (24‐Fr) was inserted after removal of the 20‐Fr gastrostomy tube.

Wandrey et al. [2] reported that the BPS‐NI score demonstrates efficacy for evaluating pain in patients outside of the intensive care unit. Chanques et al. [3] demonstrated the usefulness of the BPS‐NI for assessing pain during procedures in patients with delirium. Several reports have used the BPS‐NI to evaluate the pain associated with endoscopy. Shi et al. [4] conducted a prospective randomized clinical trial evaluating pain during colonoscopy under sedation in 225 patients, using the BPS‐NI. In another prospective study of 108 patients who underwent gastrointestinal endoscopy with sedation, Xu et al. [5] evaluated pain during endoscopy using the BPS‐NI. These reports support the use of the BPS‐NI to assess pain objectively during gastrostomy tube replacement.

The ZERO and non‐ZERO groups demonstrated no differences in background diseases, nutritional status, or confirmatory imaging used during tube replacement. Thus, no other significant differences, other than whether the ZERO was used, contributed to differences in the three BPS‐NI scores during gastrostomy tube replacement. In the study by Wandrey et al. [2], a BPS‐NI score of 4 points indicated moderate pain, while a score of 5 points indicated severe pain. Gerald et al. [3] reported that the BPS‐NI score at rest in 30 non‐intubated patients unable to self‐report their pain was 3.0 (IQR: 3.0–3.8). In our study, the median BPS‐NI scores during tube removal or insertion were 3.0 in the ZERO group, suggesting that the patients felt almost no pain.

We demonstrated that inserting the ZERO (24‐Fr) into a 20‐Fr fistula caused significant pain, an issue that should be investigated with the increased use of the ZERO in the future. For a 20Fr (6.7 mm) fistula, the bumper diameter of the non‐ZERO 20Fr is 11–12 mm even when stretched and narrowed. Despite the ZERO tip being clearly thinner than the non‐ZERO with a 9.3 mm capsule diameter, it caused significant pain during tube replacement. It is unclear why the ZERO did not reduce pain in cases where the fistula diameter was smaller than the capsule. The hardness or shape of the capsule may also be contributing factors. We hope that the ZERO suitable for a 20Fr fistula will be developed. This study has some limitations. First, this was a single‐center retrospective study; thus, further multicenter prospective studies are required to evaluate the generalizability of our results. Second, the sample size was relatively small. However, despite the small sample size, this study has high clinical value, as it provided evidence demonstrating the effectiveness of the ZERO, which had not been revealed previously. Third, it is questionable whether pain assessment using BPS‐NI is valid in patients with cerebrovascular diseases, neurodegenerative diseases, traumatic brain injury, or dementia, who commonly undergo PEG. Although PAIN‐AD has been shown to be useful in patients with dementia [2], we found it difficult to assess consolability in a very brief procedure such as tube replacement, so we used the simpler BPS‐NI. Gerard et al. reported that the BPS‐NI was effective for assessing pain in delirium patients [3], and it may be useful for a wide range of disorders, including dementia. Further research is needed to enhance the reliability of the BPS‐NI.

Based on the results of this study, one recommendation for addressing patients who report pain during the procedure is to replace existing tubes with the ZERO; additionally, the ZERO may be useful for gastrostomy tube replacement outside of hospitals. Other bumper‐type gastrostomy tubes are reported to be associated with pain and bleeding during replacement [6, 7], while balloon‐type tubes are commonly used in non‐hospital settings. The new bumper‐type ZERO allows for replacement with minimal physical resistance, similar to balloon‐type tubes, and has the advantage of requiring replacement less often than do balloon‐type tubes.

## 5 Conclusion

This study evaluated the efficacy of a new bumper‐type gastrostomy tube, the ZERO, in terms of pain during tube insertion or removal. We revealed that the ZERO was associated with significantly reduced pain as compared to non‐ZERO gastrostomy tubes. Further research is required to verify the efficiency of the ZERO in larger sample sizes.

## Author Contributions

**Kazumi Shimamoto** designed the study, conceived the main ideas, and prepared the proof. Hiromitsu Ban contributed significantly to data analysis and interpretation. **Kazumi Shimamoto** and **Hiromitsu Ban** contributed to the final version of this manuscript. **Kazumi Shimamoto** and **Masanori Hongo** collected data. **Naoki Dan**, **Yu Kobayashi**, and **Hiromitsu Ban** contributed to data analysis. All authors critically revised the manuscript, commented on drafts of the manuscript, and approved the final report.

## Funding

This research did not receive any specific grants from funding agencies in the public, commercial, or not‐for‐profit sectors.

## Conflicts of Interest

The authors declare no conflicts of interest.

## Ethics Statement

All experimental protocols described in this study were approved by the Institutional Ethical Review Committee of the Omi Medical Center (authorization number: 2025‐0055). The study adhered to the ethical guidelines of the Japan Ministry of Health, Labor and Welfare for Medical and Health Research Involving Human Subjects, and conformed to the principles outlined in the Declaration of Helsinki.

## Consent

The opt‐out recruitment method was used, allowing patients to decline participation; informed consent was thus obtained from all participants.

## Clinical Trial Registration

N/A

## Supporting information




**Supporting Table 1**: BPS‐NI, Non‐intubated Behavioral Pain Scale
