# Supplementary material for: Comparison of the Non‐intubated Behavioral Pain Scale Score During Replacement of Ideal Button ZERO and Non‐ZERO Gastrostomy Tubes
Source: DEN Open. 2025 Nov 27;6(1):e70254. doi: 10.1002/deo2.70254 (PMC12660045; doi:10.1002/deo2.70254)
Supplement: Supplementary file 1 — Supporting Table 1: BPS‐NI, Non‐intubated Behavioral Pain Scale [file DEO2-6-e70254-s001.docx]

**Table S1**. Non-Intubated Behavioral Pain Scale (BPS-NI)

| **Item** | **Description** | **Score** |
| --- | --- | --- |
| Facial expression | Relaxed | 1 |
|  | Partially tightened (brow lowering) | 2 |
|  | Fully tightened (eyelid closing) | 3 |
|  | Grimacing (cheek folded) | 4 |
| Upper or lower limbs | No movement | 1 |
|  | Partially bent upper or lower limbs | 2 |
|  | Fully bent with finger flexion | 3 |
|  | Permanently retracted, affecting inspection | 4 |
| Vocalization | No pain vocalization | 1 |
|  | Moaning not frequent (≤ 3/min) or prolonged (≤ 3 s) | 2 |
|  | Moaning frequent (≥ 3/min) or prolonged (≥3 s) | 3 |
|  | Howling or verbal complaint including “ow,” “ouch” | 4 |
